# Accuracy of the shock index and various modified shock indexes to predict early mortality in patients suffering from gastrointestinal haemorrhage

**DOI:** 10.1186/cc12157

**Published:** 2013-03-19

**Authors:** J St-Cyr Bourque, J Cliche, J Chauny, R Daoust, J Paquet, É Piette

**Affiliations:** 1 Hôpital du Sacré-Coeur de Montréal, Canada

## Introduction

The shock index (SI) is an easy-to-use clinical tool that rapidly identifies patients at risk of haemodynamic decompensation. Previous studies focused primarily on patients suffering from pneumonia, pulmonary embolism, ruptured ectopic pregnancy and traumatic haemorrhagic shock. Modified SIs have also been studied. Could the SI be accurate in patients suffering from gastrointestinal (GI) haemorrhage? The aim of this study was to compare the performance of the SI with various modified SIs and conventional vital signs in predicting 30-day mortality in a population of patients with a GI haemorrhage.

## Methods

A single-center *post-hoc *analysis was conducted of prospectively collected data from patients diagnosed with a GI haemorrhage episode in an academic emergency department (ED) from March 2008 to December 2011. Data were extracted from two databases used at our ED. The SI (pulse/systolic blood pressure) and nine modified SIs were calculated from the available first documented vital signs. ROC curves were used to determine sensitivity and specificity of the different SIs in predicting 30-day mortality.

## Results

Of the 770 patients included in the analysis, 52 died within 30 days. The standard SI at a cutoffpoint of 0.7 had the highest predictability and sensitivity of 30-day mortality (area under the curve (AUC) = 0.7, sensitivity = 0.79, specificity = 0.56). In comparison, one of the modified SIs (pulse/diastolic blood pressure) had 0.65 sensitivity and 0.71 specificity (AUC = 0.73). A heart rate >100 bpm predicted 30day mortality with 0.40 sensitivity and 0.82 specificity (AUC = 0.63).

## Conclusion

To our knowledge, this is the first study to examine the relationship between the SI and mortality in patients with a GI haemorrhage. It appears that the standard SI, when compared with various modified SIs and conventional vital signs, had the highest combined predictability and sensitivity of 30-day mortality in a population of patients suffering from GI haemorrhage. Further prospective studies are needed to confirm these findings.

